# The German research consortium for the study of bipolar disorder (BipoLife): a magnetic resonance imaging study protocol

**DOI:** 10.1186/s40345-021-00240-6

**Published:** 2021-11-17

**Authors:** Christoph Vogelbacher, Jens Sommer, Verena Schuster, Miriam H. A. Bopp, Irina Falkenberg, Philipp S. Ritter, Felix Bermpohl, Catherine Hindi Attar, Lisa Rauer, Karolin E. Einenkel, Jens Treutlein, Oliver Gruber, Georg Juckel, Vera Flasbeck, Christoph Mulert, Martin Hautzinger, Andrea Pfennig, Silke Matura, Andreas Reif, Dominik Grotegerd, Udo Dannlowski, Tilo Kircher, Michael Bauer, Andreas Jansen

**Affiliations:** 1 Department of Psychiatry and Psychotherapy, University of Marburg, Rudolf-Bultmann-Straße 8, 35039 Marburg, Germany; 2 Center for Mind, Brain, and Behavior (CMBB), Universities of Marburg and Gießen, Marburg, Germany; 3 Core-Facility Brainimaging, Faculty of Medicine, University Marburg, Marburg, Germany; 4 Department of Neurosurgery, University Marburg, Marburg, Germany; 5 Department of Psychiatry and Psychotherapy, University Hospital Carl Gustav Carus, Technische Universität Dresden, Dresden, Germany; 6 Charité – Universitätsmedizin Berlin, corporate member of Freie Universität Berlin, Humboldt-Universität zu Berlin, and Berlin Institute of Health, Department of Psychiatry and Psychotherapy, Charité at St. Hedwig-Krankenhaus, Berlin, Germany; 7 Section for Experimental Psychopathology and Neuroimaging, Department of General Psychiatry, Heidelberg University, Heidelberg, Germany; 8 Department of Psychiatry, Psychotherapy and Preventive Medicine, LWL University Hospital, Ruhr University Bochum, Bochum, Germany; 9 Center of Psychiatry, Justus-Liebig University, Giessen, Germany; 10 Giessen Graduate School for Life Sciences, Justus-Liebig University, Giessen, Germany; 11 Department of Psychology Clinical Psychology and Psychotherapy, Eberhard Karls University, Tübingen, Germany; 12 Department of Psychiatry, Psychosomatic Medicine and Psychotherapy, University Hospital Frankfurt, Frankfurt, Germany; 13 Department of Psychiatry, University of Münster, Münster, Germany

**Keywords:** BipoLife, Bipolar disorder, Major depression, fMRI, MRI quality assurance, Multicenter study, Early recognition, Early intervention

## Abstract

**Background:**

Bipolar disorder is one of the most severe mental disorders. Its chronic course is associated with high rates of morbidity and mortality, a high risk of suicide and poor social and occupational outcomes. Despite the great advances over the last decades in understanding mental disorders, the mechanisms underlying bipolar disorder at the neural network level still remain elusive. This has severe consequences for clinical practice, for instance by inadequate diagnoses or delayed treatments. The German research consortium *BipoLife* aims to shed light on the mechanisms underlying bipolar disorders. It was established in 2015 and incorporates ten university hospitals across Germany. Its research projects focus in particular on individuals at high risk of bipolar disorder, young patients in the early stages of the disease and patients with an unstable highly relapsing course and/or with acute suicidal ideation.

**Methods:**

Functional and structural magnetic resonance imaging (MRI) data was acquired across nine sites within three different studies. Obtaining neuroimaging data in a multicenter setting requires among others the harmonization of the acquisition protocol, the standardization of paradigms and the implementation of regular quality control procedures. The present article outlines the MRI imaging protocols, the acquisition parameters, the imaging paradigms, the neuroimaging quality assessment procedures and the number of recruited subjects.

**Discussion:**

The careful implementation of a MRI study protocol as well as the adherence to well-defined quality assessment procedures is one key benchmark in the evaluation of the overall quality of large-scale multicenter imaging studies. This article contributes to the *BipoLife* project by outlining the rationale and the design of the MRI study protocol. It helps to set the necessary standards for follow-up analyses and provides the technical details for an in-depth understanding of follow-up publications.

**Supplementary Information:**

The online version contains supplementary material available at 10.1186/s40345-021-00240-6.

## Background

Bipolar disorder (BD) is a severe, recurrent and heterogeneous mental disorder that affects more than 1% of the population worldwide. It usually has its onset during youth. Its chronic course is associated with high rates of morbidity and mortality, a high risk of suicide, and poor social and occupational outcomes, making it one of the main causes of disability among young and working-age people worldwide (Murray 1996). BD is one of the most heritable mental illnesses (McGuffin et al. 2003). Bipolar offspring are at a higher risk of developing BD than the general population (Axelson et al. 2015). There is strong evidence that the index (hypo)manic episode in both bipolar offspring and community cohorts is frequently preceded by prodromal affective or non-affective symptoms (Duffy et al. 2014). Besides the genetic predisposition, a subgroup of patients suffering from Attention Deficit Hyperactivity Disorder (ADHD), as well as patients diagnosed with a single or recurrent depression are more likely to develop BD (Duffy 2012; Pfennig et al. 2016). A first-episode BD is then followed by an unpredictable and relapsing course throughout the lifespan.

Despite the great advances over the last decades in understanding mental disorders, the mechanisms underlying BD at the cellular, transmitter and neural network level still remain elusive. This has severe consequences for clinical practice. For instance, only a subpopulation of patients shows good responses to lithium monotherapy. Clinical characteristics alone are often not sufficient to guide an effective choice of different treatment options. Therefore, more research is necessary for the development of individualized treatment plans. In particular individuals at high risk of BD, young patients in the early stages of the disease, and patients with an unstable highly relapsing course and/or with acute suicidal ideation have been identified as those in need (Pfennig et al. 2020; Ritter et al. 2016).

The German research consortium *BipoLife* aims to shed light on the elusive basis and causes of BD. It was established in 2015 and incorporates ten university hospitals across Germany (Berlin, Bochum, Dresden, Frankfurt, Göttingen, Hamburg, Heidelberg, Marburg, Munich and Tübingen) as well as the medical informatics section of the University of Göttingen. Its integrated multi-center approach focuses on the prevention, diagnosis and treatment of BD, as well as the identification of genomic, transcriptomic and proteomic disease markers. The major goals are the development of new diagnostic approaches and innovative therapeutic interventions as well as the translation of scientific knowledge into clinical practice. The *BipoLife* network comprises two multicenter clinical studies, a naturalistic-epidemiological study, two neuroscientific research projects and two translational platforms for the application of innovative genetic and imaging methods. The scientific knowledge gained through the projects of this consortium shall be used in the long term to improve early detection and targeted early intervention, thereby reducing the individual and socio-economical burden of BD. A detailed description of the consortium and the projects can be found in (Ritter et al. 2016).

Within the research network, magnetic resonance imaging (MRI) data were acquired in three clinical subprojects (termed “project A1”, “project A2” and “project B2” in the *BipoLife* nomenclature). The same neuroimaging protocol was used in all projects. Prior to describing the imaging protocol, the clinical subprojects in which neuroimaging data were collected, will be shortly summarized (see Ritter et al. (2016) for an extensive overview).

The aim of **project A1** (“Improving early recognition and intervention in people at-risk of developing bipolar disorder”) was to evaluate and improve early recognition and intervention strategies in individuals at increased risk for developing BDs using a naturalistic, longitudinal design. The baseline assessment was followed by biannual follow-ups for a minimum of 24 months. Three groups of individuals were studied: (1) Help-seeking adolescents and young adults aged 15–35 years without a diagnosis of BD consulting early detection centers and specialized services with ≥ 1 proposed risk factor for BD; (2) In-/outpatients with a depressive episode aged 15–35 years; (3) in-/outpatients with ADHD aged 15–35 years. Over the study period, the natural course of risk and resilience factors, early symptoms of BD, and changes in symptom severity (including the transition to BD) were observed using recently developed psychometric testing. Participation in the neuroimaging assessments was optional for the participants, i.e., they could also participate in the clinical part of project A1 without being measured by MRI. Neuroimaging data were acquired only during the baseline assessment, not during the follow-up appointments. The imaging data will be used, for instance, to develop biomarkers to improve the prediction of transition or remission in distinct at-risk subgroups of BD.^1^ The clinical study protocol of project A1 is described in detail in (Pfennig et al. 2020).

**Project A2** (“Adjuvant psychotherapy in early stage bipolar disorder”) focuses on the effects of different forms of adjuvant psychotherapies for relapse prevention in BD. This randomized controlled clinical trial aims to test the hypothesis that the addition of a specific short-term emotional-cognitive psychotherapy (“Spezifische Emotional-Kognitive Psychotherapie”, SEKT) to standard psychiatric care (treatment as usual, TAU) in comparison to a supportive, self-directed therapy (“Fördernde, Emotionsfokussierte, Supportive Psychotherapie”, FEST) will lead to positive outcomes in younger patients with BD (age 18–35 years), for instance, reduced relapse rates, missed days at work/school or days spent in hospitals. As in project A1, participation in the neuroimaging assessments was optional. Neuroimaging data were acquired at two time points, i.e. prior to and after the completion of the psychotherapeutic interventions. The imaging data will be used, for instance, to test the hypothesis that specific psychotherapy (SEKT) will have a stronger effect on neural networks associated with emotion regulation and social cognition compared to FEST (Stamm et al. 2020).

**Project B2** is an add-on project for an ongoing separate multicenter, double-blind, randomized controlled trial investigating the effect of lithium in addition to TAU in affectively ill patients with acute suicidality over 5 weeks (see Lewitzka et al. 2015) for a description of the study protocol). It aims to identify neuroimaging markers that are associated with anti-suicidal lithium treatment response. Neuroimaging data were acquired at two time points, i.e. prior to and following the 5-week intervention. The imaging data will be used, for instance, to develop biomarkers to predict successful anti-suicidal treatment with lithium.

Acquiring neuroimaging data from up to nine different sites (all centers except Munich) required the harmonization of the acquisition protocol, the standardization of paradigms and the implementation of regular quality control procedures to detect signal aberrations and potential MR scanner malfunctions. *BipoLife*, therefore, included a dedicated translational imaging project (termed “TPP2” in the *BipoLife* nomenclature) to coordinate the acquisition and analysis of all neuroimaging data. In this article, the imaging protocol, the acquisition parameters, the imaging paradigms and the neuroimaging quality assessment procedures will be described in detail. This article thus summarizes the methodological background necessary to fully understand follow-up publications from the *BipoLife* consortium.

## Methods

In the methods section, we present details on the acquired cohorts (2.1), describe the imaging paradigms and the process of data acquisition (2.2), summarize the technical imaging infrastructure and MRI data acquisition parameters (2.3), and describe the quality assessment protocol for neuroimaging data (2.4) and data storage (2.5).

### Subjects

Neuroimaging data were acquired in seven study centers in projects A1 and A2 and in four centers in project B2. Table 1 lists the study centers that were involved in each project. In project A1, participants were measured only once, i.e., during the baseline assessment. In projects A2 and B2, participants were measured twice, i.e., before and after the project-specific therapy (psychotherapy in project A2, lithium therapy in project B2). Data acquisition started in October 2015 and ended for all projects in December 2020.

**Table 1:**
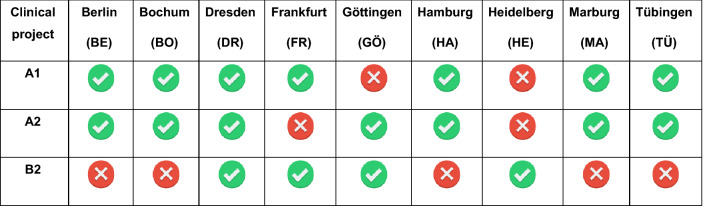
Involvement of BipoLife study centers in the neuroimaging projects A1, A2 and B2

In the following, we will describe inclusion and exclusion criteria for all subprojects. We will then present the general characteristics of the study samples. We will also present the included subjects per project to give a general overview of the acquired dataset.

**Project A1** was a naturalistic, prospective-longitudinal observational cohort study of participants aged 15–35 years. From July 2015 until September 2018, help-seeking adolescents and young adults consulting early recognition centers presenting with at least one of the proposed risk factors (family history for bipolar disorder, (increasing) mood swings, subthreshold hypomanic symptomatology, sleep/rhythm disturbances, depressive syndrome) for BD (group 1) and in- and outpatients with depressive syndrome (group 2) or ADHD (group 3), respectively, were recruited at the different sites. Overall, N = 2279 persons were screened for inclusion and exclusion criteria. N = 1419 participants were included in the study. Due to their baseline diagnostic status, N = 1229 risk participants were assigned to one of the three risk groups (see Pfennig et al. (2020) for a detailed description of the study cohort). All participants received the option to participate in the MRI assessments. Overall, N = 310 participants were measured with MRI (group1: n = 123; group 2: n = 146; group 3: n = 44) (Table 2).

**Table 2:** Project A1: Subjects’ characteristics (sex, age)

	BE	BO	DR	FR	HA	MA	TÜ	Total	Sex (male/female)	Mean age (± SD)
Group 1	39	6	32	10	4	26	6	123	63/60 (51.2% / 48.8%)	24.8 (± 5.1)
Group 2	27	3	6	22	42	39	7	143	68/78 (46.6% / 53.4%)	25.4(± 5.4)
Group 3	0	0	0	17	0	15	12	44	26/18 (59.1% / 40.9%)	27.1 (± 3.9)

310 participants were assessed with fMRI in project A1. They were assigned to one of three risk groups. The present table shows how many subjects were measured in each center (*BE* Berlin, *BO* Bochum, *DR* Dresden, *FR* Frankfurt, *HA* Hamburg, *MA* Marburg, *TÜ* Tübingen)

**Project A2** was a randomized controlled clinical trial. From August 2015 until December 2019, young patients (age between 18 and 35 years) suffering from BD with at least one episode during the preceding two years were recruited. All participants had to be in stable remission and regular medical care (including mood-stabilizing medication). Patients were randomized to one of the two psychotherapies, i.e. either SEKT or FEST (see Stamm et al. (2020) for a detailed description of the study design). All participants received the option to additionally participate in the MRI assessment before and after therapy about five months later. Overall, 66 patients were measured with MRI before the start of the therapeutic intervention and 38 patients after completion of the therapeutic intervention (Table 3). Additionally, a cohort of 35 healthy control subjects matched for age, sex, education status (as assessed by the highest graduation certificate) and handedness (as assessed by the Edinburgh handedness inventory, (Oldfield 1971)) was recruited (Table 3).

**Table 3:** Project A2: Subjects’ characteristics (sex, age)

	BE	BO	DR	GÖ	HA	MA	TÜ	Total	Sex (male/female)	Mean age (± SD)
Patients, T1	22	2	9	6	12	5	9	65	36/29 (55.4% / 44.6%)	32.7 (± 9.3)
Patients, T2	15	0	7	2	5	3	6	38	21/17 (55.3% / 44.7%)	32.9 (± 8.8)
Controls, T1	21	0	0	0	2	2	10	35	15/20 (42.9% / 57.1%)	30.2 (± 8.6)
Controls, T2t	19	0	0	0	0	2	3	25	14/11 (56.0% / 44.0%)	32.0 (± 9.3)

65 patients were assessed with MRI in project A2 before therapy (T1), 38 were assessed a second time after therapy (T2). They were randomly assigned to one of two treatment groups (i.e., either SEKT or FEST). The present table shows how many subjects were measured in each center (*BE* Berlin, *BO* Bochum, *DR* Dresden, *GÖ *Göttingen, *HA* Hamburg, *MA* Marburg, *TÜ* Tübingen). Additionally, a group of healthy control subjects was measured

**Project B2** was an add-on project for an ongoing separate multicenter, double-blind, randomized controlled trial investigating the effect of lithium (see Lewitzka et al. (2015) for a description of the study protocol). The participants received the option to participate in the MRI assessments before and after therapy five weeks later. Until the end of the project, 21 patients were included before therapy, 16 after therapy (Table 4).

**Table 4:** Project B2: Subjects’ characteristics (sex, age)

	DR	FR	GÖ	HE	Total	Sex (male/female)	Mean age (± SD)
Patients, T1	19	0	1	1	21	14/7 (66.6%/33.3%)	34.0(± 12.1)
Patients, T2	15	0	1	0	16	11/5 (68.8% / 31.2%)	35.6 (± 12.7)

21 patients were assessed with MRI in project B2 before therapy (T1), 16 were assessed a second time after therapy (T2). The present table shows how many subjects were measured in each center (*DR* Dresden, *FR* Frankfurt, *GÖ* Göttingen, *HE* Heidelberg)

### Experimental design

**Fig. 1:**
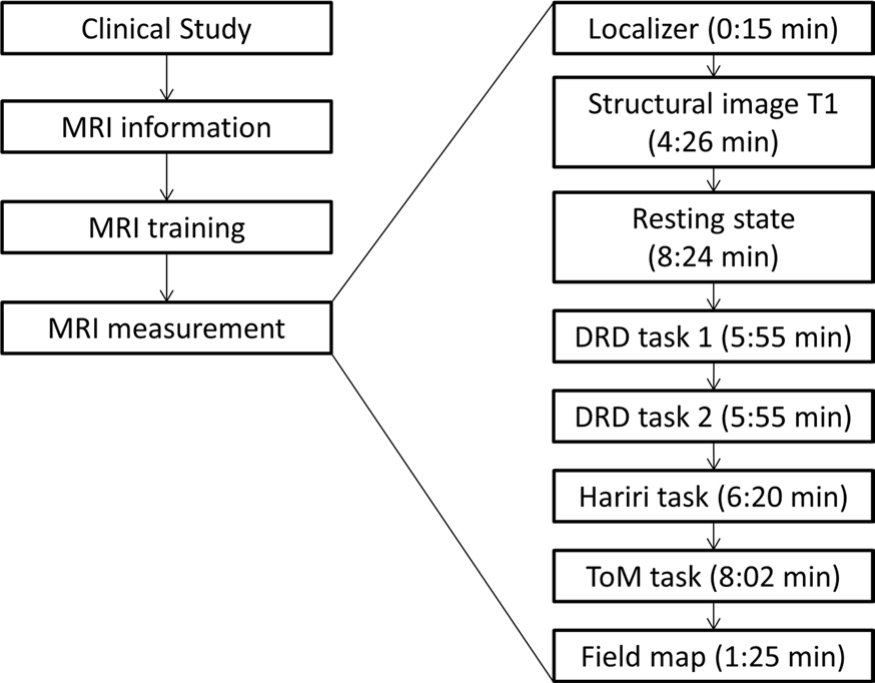
Study overview. Left: After inclusion in the clinical studies A1, A2 and B2, respectively, subjects received the option to additionally participate in the MRI assessments (“MRI information”). If they were eligible and consented, they were introduced to each task outside the MR scanner (“MRI training”). Right: The MRI measurement consisted of a localizer scan, a structural T1-weighted image, a resting state fMRI sequence, three task-based fMRI paradigms (DRD task, “Hariri” task and ToM task) and a field map. The DRD task was divided into two sessions

After inclusion in the clinical projects A1, A2 and B2, respectively, subjects received the option to additionally participate in the MRI assessments. They were included in the MRI study if they consented and if they met typical MRI safety regulations (e.g. no pacemaker). The MRI scanning protocol consisted of a structural localizer, a T1-weighted high-resolution anatomical image, a resting-state functional MRI (fMRI) sequence, three task-based fMRI paradigms, a field map and a gel-phantom measurement for quality assessment. The resting-state took 8:24 min. Participants were asked to relax, keep their eyes open and fixate on a fixation cross. The task-based paradigms assessed reward functions (“desire-reason dilemma (DRD) task”, (Diekhof et al. 2012)), emotion processing (“Hariri task”, (Dannlowski et al. 2012)), and Theory-of-Mind (ToM) functions (Walter et al. 2011). All MRI sequences were performed in the same fixed order. The MRI battery took 52 min scanning time per session (about 43 min for the measurement of the participants, 9 min for the phantom measurement). Prior to the MRI measurement, participants were introduced to each task outside the MR scanner. For each task, participants were instructed using a standardized protocol. The standardization included identical instructions through predefined texts and introduction slides, both for the training of the tasks and the actual performance in the MR scanner. Staff at each site was trained for this standardized protocol before the study started. An overview of the study course is presented in Fig. 1.

### Desire-reason dilemma (DRD) task

The DRD task used conditioned reward stimuli in different experimental situations, thereby allowing the investigation of subcortical structures of the dopaminergic reward system and their specific functional interactions with prefrontal cortices. The paradigm has been described in detail elsewhere (Diekhof et al. 2012; Diekhof and Gruber 2010).

The DRD task consisted of two parts. The first part was an operant conditioning task that was performed outside of the MR scanner. The goal of the task was the establishment of stimulus–response-reward contingencies relevant for the second part of the experiment. Subjects learned to associate specific colors and responses with either an immediate reward or a neutral outcome. The choice between a left and a right button was free. The participants were encouraged to explore the stimulus–response-reward contingencies to maximize their overall outcome. In total, squares of eight different colors were presented twenty times each in a randomized sequence. Each square was shown until the subject pressed the response button. Two of these colors led to an immediate reward (i.e., reward of 10 points) when collected with a left button press. Four colors always led to a neutral outcome regardless of button choice. The selection of the remaining two colors with a left button press led to an immediate loss (i.e., loss of 10 points). These latter two colors were included in the conditioning task to prevent a behavioral preference for the left response button also in response to the neutral colors.

After the participant was familiarized with the colors and sufficient conditioning with reward stimuli the second part (the regular task) was performed outside the scanner to train the short reaction time. The stimulus material (i.e., rewarded and neutral colors) was the same as in the first part except for the ‘potential punishment’ stimuli that were not presented. In contrast to the first part, however, participants had to pursue a superordinate long-term goal during task blocks of 4 or 8 trials. They were required to acquire 50 points for a successful completion of each task block. The superordinate goal of an individual task block was to collect the two target colors that were defined at the beginning of each block. Target colors could occur more than once within a block and had to be collected upon each appearance to reach the goal. Apart from this, subjects also had to incorporate one of two context rules into their decisions, which determined how to treat the remaining non-target colors to successfully finish a block (i.e., to gain 50 points for achievement of the superordinate long-term goal). In the “reason context”, all non-target colors had to be rejected regardless of their immediate reward association to achieve the superordinate goal. In the “desire context”, subjects were free to also collect the two conditioned (rewarding) non-target colors for an immediate bonus, whereas all remaining unrewarded non-target colors had to be rejected. Bonuses acquired in the “desire context” were added to the 50 points at the end of a block, if the long-term goal was successfully reached. Although subjects were free to decide whether to collect or to reject conditioned (rewarding) non-targets in the “desire context”, the optimal strategy for reward maximization was—apart from collecting the targets and rejecting the unrewarded non-targets—to give into the “desire” to acquire the immediately rewarded non-target colors. Conversely, during the “reason context”, subjects were forced to overcome the behavioral tendency to respond to these conditioned (rewarding) stimuli, which contradicted the superordinate long-term goal. This means that in the latter case, participants had to exert self-control to resolve this “desire-reason dilemma”.

In both the “desire” and the “reason context”, the conditioned (rewarding) non-targets, as well as the unrewarded non-targets, could occur up to 30 times each. Goal-relevant target stimuli could appear up to 60 times each in a pseudorandomized sequence with a counterbalanced trial order. Goal failures reduced these numbers because a failure to implement the long-term goal terminated a block immediately before its actual end with the feedback “goal failure”. The consequence of such a goal failure was a loss of the points already acquired within that respective task block.

**Fig. 2:**
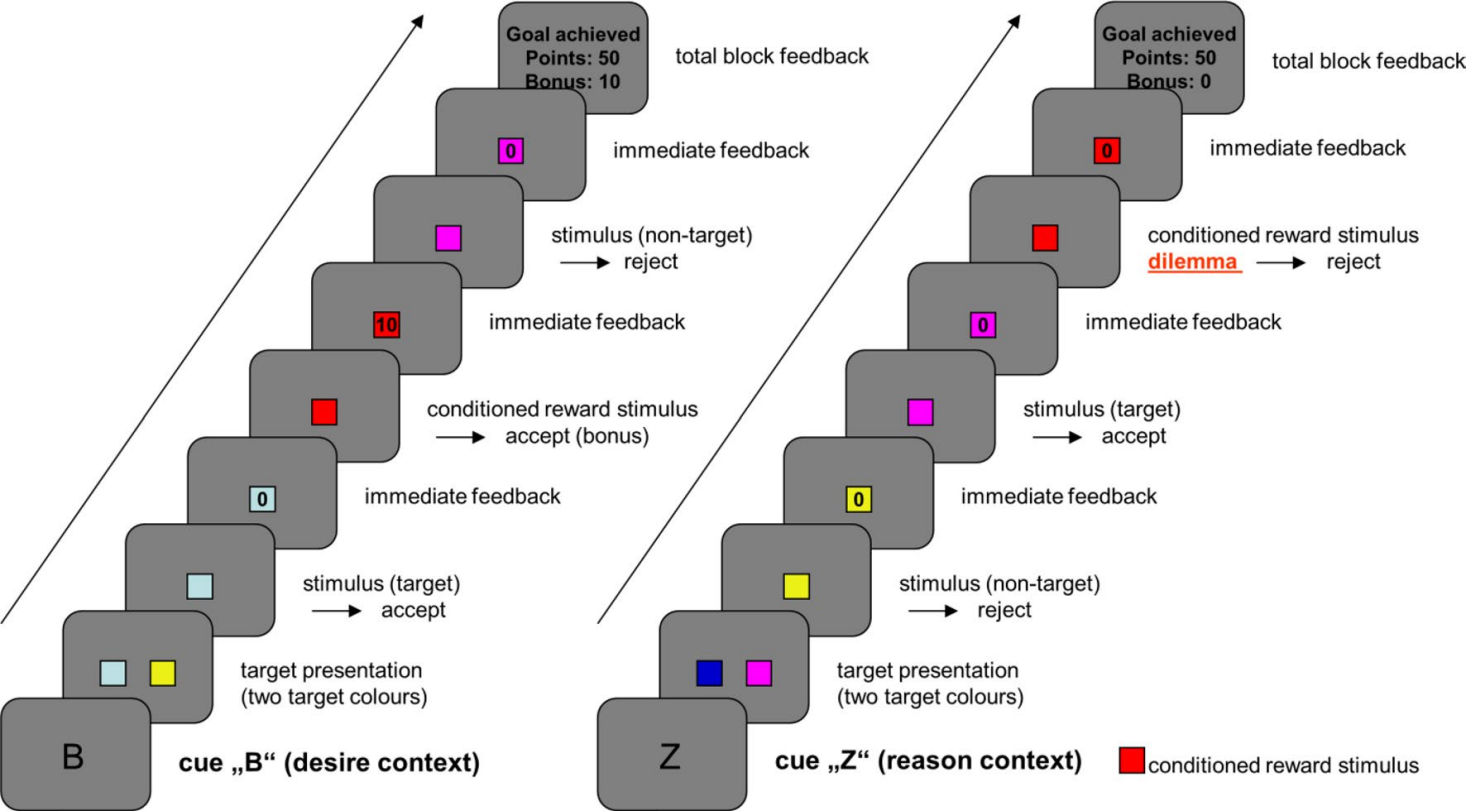
Graphical depiction of the DRD task. Subjects got first presented the cue (either the desire context (left) or the reason context (right)) and then had to react on the shown colors as priory trained

In the scanner, subjects completed 40 task blocks over the course of two fMRI runs. Half of the task blocks were performed in the “desire context”, the other half in the “reason context” (Fig. 2). The context always changed after two consecutive task blocks, which was indicated by a context cue. Context cues indicating a change in decision context always appeared for 1800 ms (followed by a 200 ms blank screen delay). Subsequently, the two relevant target colors for the upcoming task block were shown for 1500 ms (preceded by a 200 ms blank screen delay and followed by another 200 ms, in which a blank screen was presented). The relevant target colors changed every task block. The display of the two relevant target colors was followed by individual trials, in which subjects had to collect relevant targets and also bonuses in the “desire context” or only had to collect targets in the “reason context”. An individual trial had a duration of 1900 ms. It started with a grey blank screen (duration = 200 ms) before a colored square was shown for 900 ms, which was followed by immediate feedback for the current choice the subject had made. This feedback had a duration of 700 ms. A trial ended with a blank screen, which was shown for 100 ms. The total feedback, which indicated the overall outcome of a task block (including bonuses), was always presented at the end of the respective task block (for 1800 ms) and was followed by a grey blank screen of 100 ms before the next task block began or a change in context was indicated. Failure to implement the superordinate task goal or failure to answer within 900 ms led to the termination of the current task block and zero outcomes (goal failure; see also above). Points acquired in the experiment were cashed into real money. Subjects could receive up to 30 €, which were added to the general reimbursement for participation.

### Emotional face-matching task (“Hariri”)

**Fig. 3:**
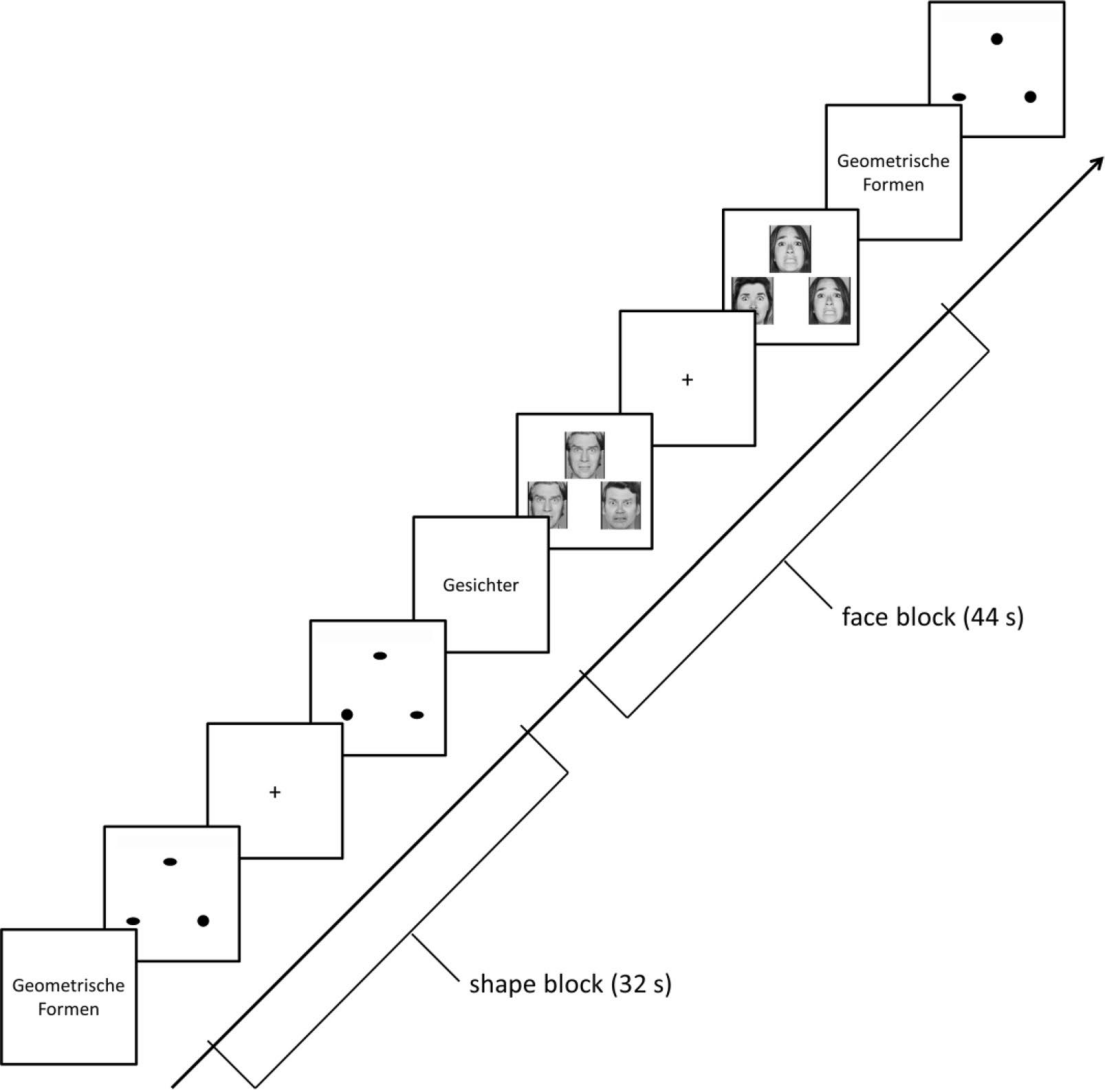
Graphical depiction of the emotional face-matching task. Subjects viewed in blocks either gray-scale images of fearful or angry faces (block length 44 s) or geometric shapes (block length 32 s). Each block started with a short introductory screen (“Geometrische Formen” = geometrical shapes; “Gesichter” = faces)

The face-matching task aimed at activating face processing regions (e.g., fusiform face area), limbic regions (e.g., amygdala) and prefrontal regions (Hariri et al. 2002). In the active condition, subjects viewed gray-scale images of fearful or angry faces (Ekman 1992), and in the control condition, they viewed geometric shapes (circles and ellipsoids). In each trial, three items were presented. A target image was located at the top, two further images on the left and right side at the bottom, whereby one of these images was identical to the target image. Every subject was first instructed outside the scanner to this task. Inside the MR scanner, the participant had to indicate which of these two images was identical to the target image by pressing a corresponding button on an MRI-compatible response pad. The task was set up as block design, with six face and shape trials, respectively, per block. Blocks had a duration of 44 s (faces) and 32 s (shapes), respectively. Five shapes blocks and four faces blocks were presented in alternating order, starting with a shapes block (Fig. 3). Blocks were separated by short inter-block-intervals (1.5–5.5 s). The paradigm lasted 6 min 14 s.

### Theory of Mind (ToM) task

**Fig. 4:**
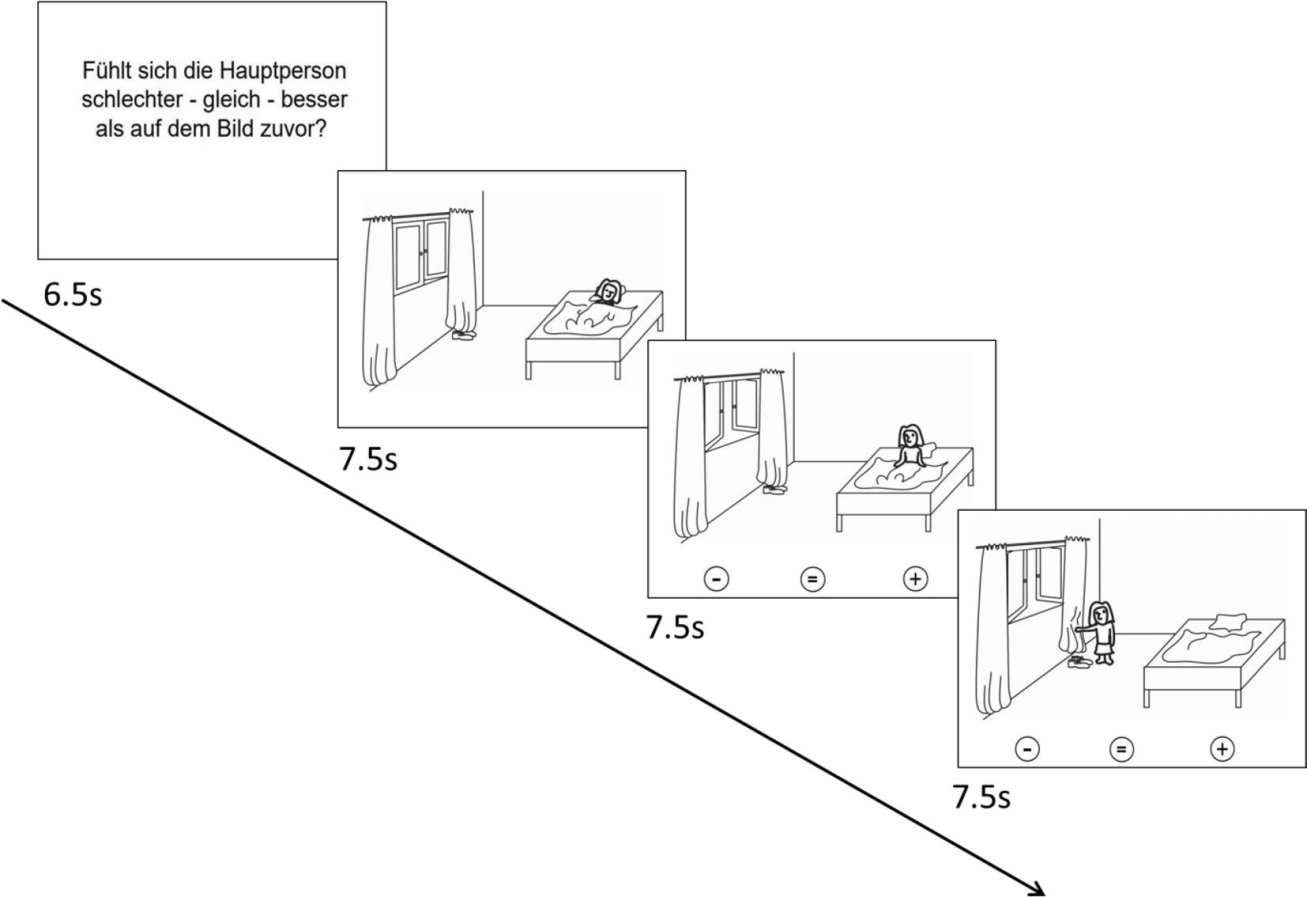
Graphical depiction of the ToM task. Each trial consisted of an introduction describing the respective task and a cartoon story consisting of three consecutive pictures. This figure illustrates the ToM condition. First an introduction text is shown “Does the person feel worse—equal—better as on the picture before”, which means that the subjects had to judge how the affective state is changed in pictures 2 and 3 compared to the previous picture. In the control condition, the subject has to judge whether the number of living beings is changed

The ToM task showed cartoons in which different social situations were depicted (see Fig. 4). It aimed to activate the ToM network relevant for social cognition since a dysfunction of the ToM network activated by this task is associated with a genetic risk variant for BD and in relatives of patients with BD (Walter et al. 2011). The paradigm has been described in detail elsewhere (Walter et al. 2011).

The ToM task consisted of two alternately presented conditions: a ToM condition and a control condition. Both conditions were presented eight times each. They started with an introduction (6.5 s) followed by a cartoon story consisting of three consecutive pictures (7.5 s per picture) (Fig. 4). All pictures were free of direct signs of the characters’ emotions (e.g., facial expressions). The subject was instructed to either evaluate in each picture the affective state of the protagonist (ToM condition) or to count the number of living beings (control condition). In the second and third picture, the subject had to indicate by a button press whether the affective state was better, equal. or worse compared to the previous picture (ToM condition) or whether the number of living beings was higher, equal or lower compared to the previous picture (ToM condition) (button press was carried out with the right hand; index finger = worse affective state / less living beings, middle finger = equal state / living beings, ring finger = better affective state, more living beings).

### MRI data acquisition parameters

All MRI data sets were acquired at 3 T MR scanners with different hardware and software configurations. An overview of the different scanner types and the receive coils is given in Table 5. Pulse sequences were first implemented and tested at the University of Marburg (which was the coordinating center). Parameters were subsequently standardized across all sites to the extent permitted by each platform. For the MR scanners from Siemens, the original MR parameters could almost all be adopted 1 to 1. However, adjustments had to be made for the Philips scanner in Bochum, which resulted in somewhat larger deviations (see Additional file 1: Appendix S1). Before the study started, scientists from the coordinating center Marburg performed site visits at all participating centers to check the adherence to the study protocol and to train the measurement procedure. Additionally, a “traveling subject” was also measured during these site-visits.

**Table 5:** List of MR scanners (manufacturer, scanner types, field strength) and receive coils at each study site

Site	Manufacturer	MRI scanner type	Field	Receive coil
Berlin	Siemens	Tim Trio	3 Tesla	20 channels
Bochum	Philips	Achieva	3 Tesla	32 channels
Dresden	Siemens	Tim Trio	3 Tesla	12 channels
Frankfurt	Siemens	Trio	3 Tesla	8 channels
Göttingen	Siemens	Tim Trio	3 Tesla	12 channels
Hamburg	Siemens	Skyra	3 Tesla	32 channels
Heidelberg	Siemens	Tim Trio	3 Tesla	12 channels
Marburg	Siemens	Tim Trio	3 Tesla	12 channels
Tübingen	Siemens	Prisma	3 Tesla	20 channels

The MRI scanning protocol consisted of a T1-weighted high-resolution anatomical image, four echo-planar imaging (EPI) sequences sensitive to blood oxygen level dependent (BOLD) contrast for fMRI measurements, a field map and an EPI measurement for quality assessment (see 2.2). The MRI sequences were always performed in the same fixed order. The T1-weighted image was used to align the measurement volumes of all the following EPI sequences. Slices were positioned transaxially parallel to the anterior–posterior commissural line (AC-PC), based on the smallest measurement volume of the sequences. This alignment was then copied to all the other sequences. Special care was taken that both temporal lobes were always included inside the measured volume.

The MR parameters of the original MR sequence are presented in Table 6. A complete list of all MRI parameters is presented for each MR scanner in Additional file 1: Appendix S1.

**Table 6:** The structural and functional MR parameters of the original MR sequence including the ranges of the parameters after the adaption to each scanner

Parameter	T1 (range)	fMRI—Resting state (range)	fMRI—DRD (range)	fMRI—Face matching (range)	fMRI—ToM (range)
Repetition time (TR)	1900 ms (shortest—1900 ms)	2000 ms	1900 ms	2000 ms	2000 ms
Echo time (TE)	2.26 ms (shortest—2.26 ms)	30 ms	30 ms	30 ms	30 ms
Field of View (FoV) mm^2^	256 × 256	210 × 210	192 × 192	210 × 210	192 × 192
Matrix size	256 × 256	64 × 64	64 × 64	64 × 64	64 × 64
Slice thickness	1.0	3.0 mm	3.0 mm	3.8	4.0 mm
Distance factor	50% (0–50%)	20%	20%	10%	25%
Flip angle	9° (8–9°)	70°	70°	90°	80°
Phase encoding direction	Anterior >> Posterior (Right > > Left)	Anterior > > Posterior	Anterior > > Posterior	Anterior > > Posterior	Anterior > > Posterior
Bandwidth	200 Hz/Px (191–200 Hz/Px)	2894 Hz/Px (2298–3987 Hz/Px)	2894 Hz/Px (2232–3653 Hz/Px)	2232 Hz/Px (2232–3987 Hz/Px)	2112 Hz/Px (2112–3653 Hz/Px)
Acquisition order	Ascending (Cartesian)	Interleaved—Ascending (Interleaved)	Interleaved—Ascending (Interleaved)	Interleaved—Ascending (Interleaved)	Interleaved—Descending (Interleaved)
Number of slices	176 (176–220)	34 (32–34)	31 (30–31)	33 (32–33)	32
Measurements		250	185	187	239
Effective voxel size (mm^3^)	1.0 × 1.0 × 1.0	3.3 × 3.3 × 3.0	3.0 × 3.0 × 3.0	3.3 × 3.3 × 3.8	3.0 × 3.0 × 4.0
Acquisition time (TA)	4:26 min (3:11–4:28 min)	8:24 min (8:24–8:37 min)	5:55 min (5:55–6:00 min)	6:20 min (6:18–6:24 min)	8:02 min (8:02–8:08 min)

### MRI Quality assessment

Large, longitudinal, multi-center MRI studies require comprehensive quality assurance (QA) protocols to assess the general quality of the acquired data, to indicate potential malfunctions in the scanning equipment and to evaluate inter-site differences that need to be accounted for in subsequent analyses. Several examples of QA protocols for MRI data are described in the literature, mostly in the context of large-scale multicenter studies (for an overview, see Glover et al. (2012), Van Horn and Toga (2009)). In the *BipoLife* study, the MR scanner characteristics were assessed by the regular measurement of a MRI phantom. Additionally, a first quality control of the human MRI data was performed using the BIDS-App *MRIQC* (Magnetic Resonance Imaging Quality Control, (Esteban et al. 2017)).

### Phantom MRI data

MR scanner characteristics were assessed by the regular measurement of a MRI phantom. The phantom was a 23.5 cm long and 11.1 cm-diameter cylindrical plastic vessel (Rotilabo, Carl Roth GmbH + Co. KG, Karlsruhe, Germany) filled with a mixture of 62.5 g agar and 2000 ml distilled water. Phantoms were built at the University of Marburg and sent to each participating center. All study sites, therefore, used the same type of phantom.

Phantom data were acquired after the measurement of each subject. The alignment of the phantom was lengthwise, i.e., parallel to the main scanner axis, and at the center of the head coil. The measurement volume was manually centered at the phantom with a slice direction perpendicular to the phantom body (see Vogelbacher et al. (2018) for a graphical depiction). We developed a QA program that focused on the temporal stability of the MRI data. Temporal stability is in particular important for fMRI measurements in which MR scanners are typically highly stressed. Therefore, the MRI phantom was measured with an EPI sequence. The same sequence parameters were chosen for the resting-state measurement. Also, the same scanner-specific reconstruction methods were employed.

**Fig. 5:**
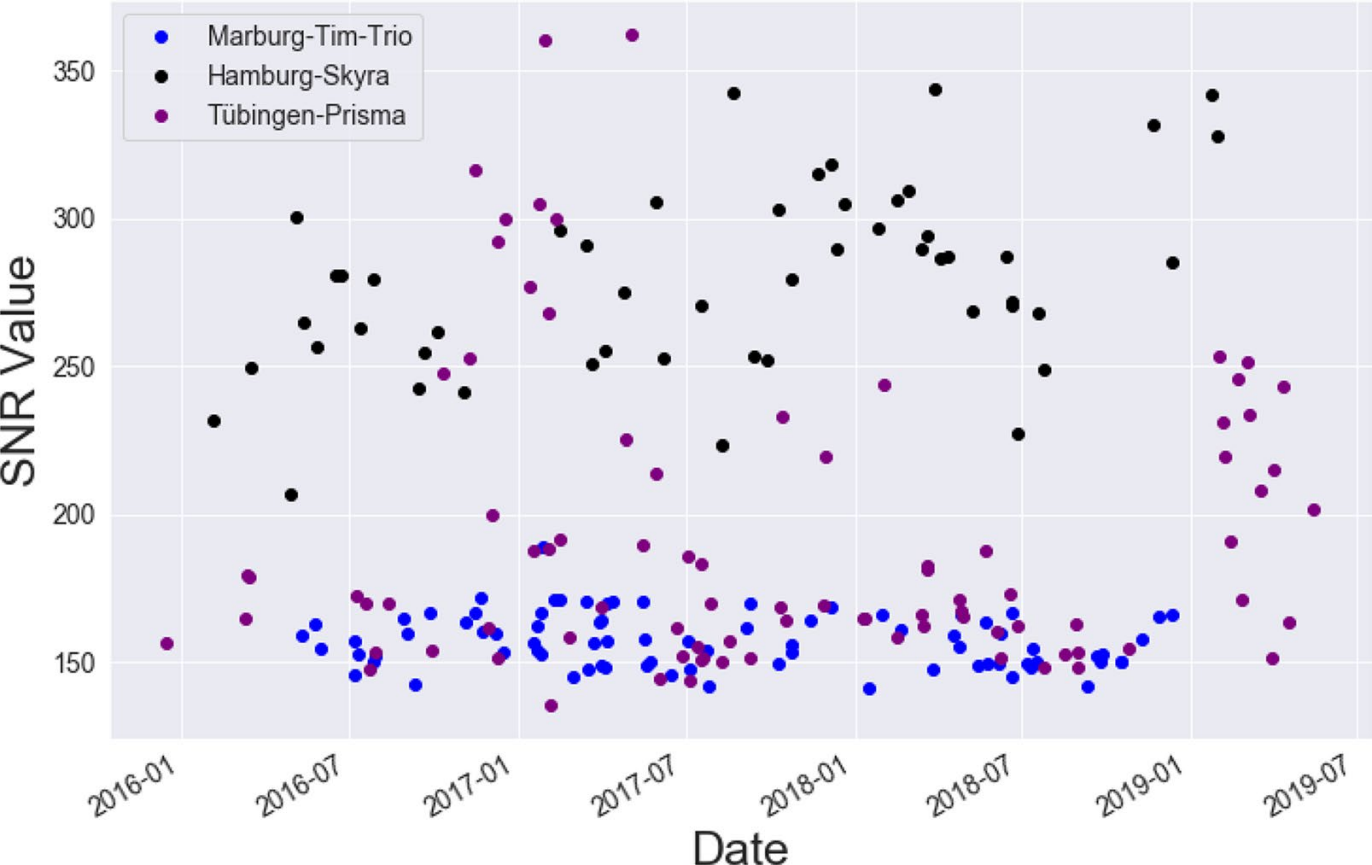
Signal-to-Noise Ratio (SNR) values of phantom measurements of three different BipoLife sites across time. The MR scanner of Marburg (Siemens Tim Trio, blue) shows stable values, but a lower SNR value compared to Hamburg (Siemens Skyra, black) or Tübingen (Siemens Prisma, purple)

A variety of QA parameters can be calculated from phantom data, for instance, geometric accuracy, contrast resolution, ghosting level, spatial uniformity and signal-to-noise ratio. The QA protocol used statistics that are described in detail in previous publications of our research groups (Vogelbacher et al. 2018). The phantom data were analyzed using the *LAB-QA2GO* software package (Vogelbacher et al. 2019). In Fig. 5, we exemplarily present the signal-to-noise ratio (SNR) values of phantom measurements of three different *BipoLife* sites across the course of the study. The MR scanner of Marburg (Siemens Tim Trio) has lower SNR values compared to the MR scanner used in Hamburg (Siemens Skyra) or Tübingen (Siemens Prisma). This is explained by the more modern and efficient technical design of the latter scanners. The Siemens Prisma is for instance the successor model of the Siemens Tim Trio (see Vogelbacher et al. (2018) for a detailed comparison of the technical performance of both MR scanners). In contrast, the variations in SNR are considerably lower for the Siemens Tim Trio. This is, however, most likely not caused by MR scanner characteristics. In Marburg, we used a self-built Styrofoam phantom holder to reduce spatial variance related to different placements of the phantom in the scanner and to decrease the time-consuming alignment procedure. This ensured that always the same part of the phantom is measured, leading to a reduction in the variance of almost all QA statistics (see Vogelbacher et al. (2018) for an extensive discussion). Such a phantom holder was not available at the other participating sites. A detailed analysis of the QA phantom data, in particular concerning the effect of different MR scanners and the impact of hardware and software changes, is described elsewhere (Vogelbacher et al., in preparation; but also see Vogelbacher et al. (2018) for a similar analysis on data from a bi-center MRI study performed by our research groups).

#### Human MRI data

Each data set was checked for completeness, both with regard to the MRI data and the corresponding log files. A frequent error source was the wrong alignment of the measurement volume in the functional measurements. Therefore, all MRI data was checked promptly to be able to give early feedback to the respective study sites. A first quality control was performed using the BIDS-App *MRIQC* (Magnetic Resonance Imaging Quality Control, (Esteban et al. 2017)). *MRIQC* assesses both structural T1-weighted MR images and BOLD-images of the brain by calculating a set of quality measures from each image. MRIQC uses 14 Image Quality Metrics that characterize each image in 56 features. The tool also includes a visual reporting system to manually investigate potential quality issues in single subjects. This information was provided to researchers who performed the subsequent data analysis.

### Data storage

After an initial quality check by staff from the local study sites, all MRI data (except for project B2) was transferred via the Internet in raw Digital Imaging and Communications in Medicine (*DICOM)* format to the coordinating center Marburg. Project B2 was an add-on project to an ongoing separate multicenter, double-blind, randomized controlled trial. The project-specific data policy did not allow data collection in Marburg. MRI data for this project was therefore stored on DVDs and sent to the University of Heidelberg. According to the study protocol of B2, the University of Heidelberg is responsible for the data storage and data analysis.

At the University of Marburg, the data was transformed from the *DICOM* format to Brain Imaging Data Structure (*BIDS)* format using *heudiconv* (Gorgolewski et al. 2016). *Heudiconv* is a flexible *DICOM* converter for organizing brain imaging data into structured directory layouts (Halchenko et al. 2020). The BIDS format has been developed to standardize data storage for neuroimaging experiments. This format enables the usage of different BIDS-Apps to analyze the imaging data with standardized software packages (e.g. fMRIPrep (Esteban et al. 2019) for a standardized preprocessing, or Statistical Parametric Mapping (SPM, https://www.fil.ion.ucl.ac.uk/spm/) to run the 1st level analysis). It also enables other scientists to access the data for different analysis approaches. We decided against centralized data analysis, but instead provided the MRI data in both DICOM and BIDS format with the additional quality information. This decision was ultimately based on practical reasons, as the funds allocated to the project were, as is often the case, available only for data collection and a first data analysis. The data will be analyzed modality-specifically by different participating centers and provided to the other centers in final form upon request.

## Discussion

The research consortium *BipoLife* is a German-wide network of university centers that aims to investigate bipolar disorder, in particular concerning its early recognition, reliable diagnosis, rational treatment and prognosis (Ritter et al. 2016). The network comprised several clinical and neuroscientific studies. MRI data were acquired in three projects. Data acquisition started in 2015 and ended in December 2020. The same neuroimaging protocol was used in all projects.

### Implementation of a multicenter study protocol

Acquiring neuroimaging data over several years from up to nine different sites requires, among others, the harmonization of the acquisition protocol, the standardization of paradigms and the implementation of regular quality control procedures. The main challenge of the present study was the implementation of a comprehensive multicenter study protocol including a QA protocol for assessing the general quality of the compiled MRI data.

The study protocol was first established at the coordinating center Marburg. Here, we implemented the various task-based fMRI paradigms, chose specific MR sequences, defined a standardized imaging workflow and acquired pilot data to ensure that the data was of sufficient quality (e.g., by checking the results of the fMRI paradigms). We also acquired the approval of the local ethics committee. In a second step, we collected systematically information on the available hardware in the other participating centers (e.g., MR scanner, available head coils, stimulation equipment), determined the personnel responsible for the set-up, provided all details on the study protocol (including MR sequence parameters, fMRI paradigms and information on the QA protocol) and established a data transfer protocol in compliance with data protection regulations. Information of the ethics proposal as well as the positive approval from Marburg was provided to all centers so that they could also get a positive vote. In a third step, a team of the coordinating center Marburg visited all participating sites, trained the local team members and verified the correct compliance with the protocol. After the final implementation, each center was asked to measure three healthy control subjects to get routine in following the study protocol. This pilot data was transferred to Marburg and checked for potential errors in data acquisition, both with regard to technical and organizational aspects (e.g., correct alignment of measurement volumes). After this check, the local center got the permission to acquire data for the BipoLife project.

A major challenge for the successful implementation of the study protocol and even more for its adherence over several years was the establishment of professional communication structures. Each center had to name responsible contact persons. If new colleagues entered the project or, even more importantly, if the main contact person changed, a comprehensive familiarization with all procedures had to be ensured. Another important aspect here was the employees' self-motivation. When colleagues identified with the project, data collection often went orders of magnitude better than when the respective contact persons were only assigned to this task by their superiors. For long-term success, it was therefore important to provide sufficient incentives for all participants (e.g., through their own research interest). In our experience, it was ultimately not so much technical aspects that were important for success, but rather organizational issues.

### Continuous assessment of data quality

Modern MR scanners have in general good technical quality, for instance high SNR, sufficient image homogeneity and good image contrast. Image characteristics, however, will inevitably change over the course of a study. This is in particular a challenge for fMRI data because functional signal changes are typically just in the order of about 1–5% of the raw signal intensity. The temporal stability of MRI acquisitions is therefore important to differentiate between MR signal changes associated with an altered disease status (e.g. by the effect of a psychotherapeutic intervention) and signal changes caused by alterations of the MR scanner system (Friedman and Glover 2006). It has been previously argued that the implementation of QA methods has become one key benchmark in the evaluation of the overall quality of large-scale imaging studies (Van Horn and Toga 2009). The documented adherence to the QA protocol will help to guide both clinicians and researchers to evaluate the quality, impact, and relevance of the study to the patient-level.

In previous large-scale multicenter studies, malfunctions of the MR scanners were, if at all, often only detected after the study was finished, for instance during a retrospective analysis of phantom data (see Friedman and Glover (2006) for an example). In the *BipoLife* consortium, the implemented QA procedures monitored MR scanner performance promptly, defined benchmark characteristics and were able to assess the impact of hard- and software changes in scanner settings. This information can be used to detect potential MR scanner malfunctions (see e.g. Friedman and Glover (2006), Vogelbacher et al. (2018) for specific examples). In the present study, however, there were no deviations indicating major deterioration in MR scanner quality. The QA information can also be implemented in subsequent MRI data analyses, for instance by excluding data from specific subjects or imaging sites or by implementing corrections for site-specific effects.

Based on prior work, it is well known that there can be large differences in various technical parameters between MR scanners (Vogelbacher et al. 2018). In the present article, we exemplary showed differences between the MR scanners in Marburg (Siemens Tim Trio), Hamburg (Siemens Skyra) and Tübingen (Siemens Prisma) by the analysis of the SNR value for the phantom data (see Fig. 5). It is evident that the more modern MR scanners (Prisma, Skyra) show a higher SNR value (Siemens Prisma is for instance the successor model of the Siemens Tim Trio). Interestingly, they also showed a higher variation of the SNR value. The latter, however, can be attributed to the analysis routine of the phantom data. For the phantom data, the middle slice of the measured volume is used (see Vogelbacher et al. (2018) for more details). In Marburg, we used a self-built Styrofoam phantom holder to reduce spatial variance related to different placements of the phantom in the scanner and to decrease the time-consuming alignment procedure. Also a fixed protocol for positioning the measurement volume was used. This ensured that always the same part of the phantom is measured, leading to a reduction in the variance of almost all QA statistics. This phantom holder was not available at other centers so that the same positioning of the phantom was not guaranteed. Internal structures of the gel phantom (e.g. air bubbles) have as well an effect to the calculation of the QA metrics.

### Data analysis

In the present description of the study protocol, we deliberately refrained from specifying more detailed data analysis pipelines for the human MRI data. On the one hand, these analyses have been already described in previous publications of the participating research groups (e.g., Dannlowski et al. (2012); Diekhof and Gruber (2010); Kessler et al. (2020); Meller et al. (2020); Nenadic et al. (2015); Trost et al. (2014); Walter et al. (2011)). On the other hand, they will be most likely adapted to the specific research questions. All MRI data (except for project B2 due to issues of protection of data privacy) was stored centrally in the recently developed BIDS format and had been checked for completeness and overall quality.

For the analysis of the human data the MRIQC tool was used for a first quality check. All reports were checked manually by the coordinating center with regard to noise level, movement, artifacts and correct positioning of the volume. The global impression and the rating of the image (excellent, acceptable, poor and exclude) were provided for each analysis so that no extensive preparatory work is needed. For each analysis an individual decision has to be made which data will be included.

### Outlook to clinical projects

Another strength of the overall study results from the combination of well-planned clinical projects with the extensive deep-phenotyping MRI approach. *Project A1* is the first investigation of a help-seeking cohort of at-risk persons for BD. Its major strengths are the large sample size and the high number of assessment points during the particularly vulnerable phase of disease development and potential conversion to BD (at least five assessments over a period of at least two years; see Pfennig et al. (2020) for an in-depth description of the study design of A1). Clinical and MRI data can be combined for instance to test whether neuroimaging data will improve the prediction of whether or not an individual will develop BD. The MRI data can be ultimately used to improve evidence-based guidelines for early detection and interventions for people at high risk for BD. *Project A2* is to our knowledge the biggest multicenter randomized controlled trial (RCT) in the field of psychotherapy in bipolar disorders to date (see Stamm et al. (2020) for a detailed study description). By comparing two different treatment approaches in the early stage of bipolar disorder, it is possible to assess to what extent a specific, cognitive behavioral therapy could be superior to a supportive, experience- and emotion-focused intervention. Clinical and MRI data can now be combined to identify potential neurobiological predictors for (successful) response to psychotherapeutic interventions in bipolar disorder. *Project B2* is an add-on imaging project for an already ongoing multicenter, double-blind RCT. It investigates the additional effect of lithium in affectively ill patients with acute suicidality, in addition to “treatment as usual” (see Lewitzka et al. (2015) or a description of the study protocol). The MRI data can be used to identify neuroimaging markers that predict successful anti-suicidal treatment with lithium. A potential limitation of this project is the relatively low number of recruited subjects. Originally, we planned to include n = 80 subjects. We were finally successful to include n = 21 for the first measurement, n = 16 for the second measurement. The main reason for this low enrollment number was the linkage to an already ongoing RCT and thus a more difficult recruitment situation.

### Summary

In summary, the present article described the MRI study protocol of the *BipoLife* project. On the one hand, it provided the technical background necessary for an in-depth understanding of the subsequent data analyses. On the other hand, it outlined how clinical and neuroimaging data can be combined to further understand the mechanisms underlying BD.

## Supplementary Information

**Additional file 1: Appendix S1.** Magnet Resonance Imaging protocol sequences of each center.

## Data Availability

Data sharing is not applicable to this article as it is a study protocol and no datasets were analyzed at this stage of the study process. When publishing the data, the datasets used and/or analyzed during the study will be available from the corresponding author on reasonable request.

## Abbreviations

AC-PC: Anterior–posterior commissure
ADHD: Attention Deficit Hyperactivity Disorder
BD: Bipolar disorder
BIDS: Brain Imaging Data Structure
BOLD: Blood oxygen level dependent
DICOM: Digital Imaging and Communications in Medicine
DRD: Desire-reason dilemma
EPI: Echo-planar imaging
FEST: An Fördernde, Emotionsfokussierte, Supportive Psychotherapie”, supportive, self-directed therapy
fMRI: Functional magnetic resonance imaging
MR: Magnetic resonance
MRI: Magnetic resonance imaging
MRIQC: Magnetic Resonance Imaging Quality Control
QA: Quality assurance
RCT: Randomized controlled trial
SEKT: “Spezifische Emotional-Kognitive Psychotherapie”, short-term emotional-cognitive psychotherapy
SNR: Signal-to-Noise ratio
TAU: Treatment as usual
ToM: Theory-of-Mind
TPP2: Translational imaging platform

## Footnotes

1 The study protocol would of course benefit from a more explicit description of the clinical hypotheses in relation to the neuroimaging data. These hypotheses were, however, neither explicitly described in the original grant proposal nor in any other publication. We therefore refrain from post-hoc specifying any hypotheses for projects A1, A2 and B2 in this protocol.
