# Supplementary material for: The German research consortium for the study of bipolar disorder (BipoLife): a magnetic resonance imaging study protocol
Source: Int J Bipolar Disord. 2021 Nov 17;9:37. doi: 10.1186/s40345-021-00240-6 (PMC8595454; doi:10.1186/s40345-021-00240-6)
Supplement: Supplementary file 1 — Additional file 1: Appendix S1. Magnet Resonance Imaging protocol sequences of each center. [file 40345_2021_240_MOESM1_ESM.docx]

# Supplemental Material - Magnet Resonance Imaging protocol sequences

## Structural image - T1 weighted

| **Site** | **Berlin** | **Bochum** | **Dresden** | **Frankfurt** | **Göttingen** | **Heidelberg** | **Hamburg** | **Marburg** | **Tübingen** |
| --- | --- | --- | --- | --- | --- | --- | --- | --- | --- |
| **Repetition time (TR)** | 1900 ms | shortest | 1900 ms | 1900 ms | 1900 ms | 1900 ms | 1900 ms | 1900 ms | 1900 ms |
| **Echo time (TE)** | 2.26 ms | shortest | 2.26 ms | 2.26 ms | 2.26 ms | 2.26 ms | 2.26 ms | 2.26 ms | 2.26 ms |
| **Field of View (FoV) mm²** | 256 x 256 | 256 x 256 | 256 x 256 | 256 x 256 | 256 x 256 | 256 x 256 | 256 x 256 | 256 x 256 | 256 x 256 |
| **Matrix size** | 256 x 256 | 256 x 256 | 256 x 256 | 256 x 256 | 256 x 256 | 256 x 256 | 256 x 256 | 256 x 256 | 256 x 256 |
| **Slice thickness mm** | 1.0 | 1.0 | 1.0 | 1.0 | 1.0 | 1.0 | 1.0 | 1.0 | 1.0 |
| **Distance factor** | 50 % | 0 % | 50 % | 50 % | 50 % | 50 % | 50 % | 50 % | 50 % |
| **Flip angle** | 9° | 8° | 9° | 9° | 9° | 9° | 9° | 9° | 9° |
| **Phase encoding direction** | Anterior >> Posterior | Right >> Left | Anterior >> Posterior | Anterior >> Posterior | Anterior >> Posterior | Anterior >> Posterior | Anterior >> Posterior | Anterior >> Posterior | Anterior >> Posterior |
| **Bandwidth** | 200 Hz/Px | 191 Hz | 200 Hz/Px | 200 Hz/Px | 200 Hz/Px | 200 Hz/Px | 200 Hz/Px | 200 Hz/Px | 200 Hz/Px |
| **Acquisition order** | Ascending | Cartesian | Ascending | Ascending | Ascending | Ascending | Ascending | Ascending | Ascending |
| **Number of slices** | 176 | 220 | 176 | 176 | 176 | 176 | 176 | 176 | 176 |
| **Effective voxel size (mm³)** | 1.0 x 1.0 x 1.0 | 1.0 x 1.0 x 1.0 | 1.0 x 1.0 x 1.0 | 1.0 x 1.0 x 1.0 | 1.0 x 1.0 x 1.0 | 1.0 x 1.0 x 1.0 | 1.0 x 1.0 x 1.0 | 1.0 x 1.0 x 1.0 | 1.0 x 1.0 x 1.0 |
| **Acquisition time (TA) min** | 4:26 | 3:11 | 4:26 | 4:28 | 4:26 | 4:26 | 4:26 | 4:26 | 4:26 |

## Functional measurement - Resting state

| **Site** | **Berlin** | **Bochum** | **Dresden** | **Frankfurt** | **Göttingen** | **Heidelberg** | **Hamburg** | **Marburg** | **Tübingen** |
| --- | --- | --- | --- | --- | --- | --- | --- | --- | --- |
| **Repetition time (TR)** | 2000 ms | 2000 ms | 2000 ms | 2050 ms | 2000 ms | 2000 ms | 2000 ms | 2000 ms | 2000 ms |
| **Echo time (TE)** | 30 ms | 30 ms | 30 ms | 30 ms | 30 ms | 30 ms | 30 ms | 30 ms | 30 ms |
| **Field of View (FoV) mm²** | 210 x 210 | 210 x 210 | 210 x 210 | 210 x 210 | 210 x 210 | 210 x 210 | 210 x 210 | 210 x 210 | 210 x 210 |
| **Matrix size** | 64 x 64 | 64 x 64 | 64 x 64 | 64 x 64 | 64 x 64 | 64 x 64 | 64 x 64 | 64 x 64 | 64 x 64 |
| **Slice thickness** | 3.0 mm | 3.0 mm | 3.0 mm | 3.0 mm | 3.0 mm | 3.0 mm | 3.0 mm | 3.0 mm | 3.0 mm |
| **Distance factor** | 20 % | 20 % | 20 % | 20 % | 20 % | 20 % | 20 % | 20 % | 20 % |
| **Flip angle** | 70° | 70° | 70° | 70° | 70° | 70° | 70° | 70° | 70° |
| **Phase encoding direction** | Anterior >> Posterior | Anterior >> Posterior | Anterior >> Posterior | Anterior >> Posterior | Anterior >> Posterior | Anterior >> Posterior | Anterior >> Posterior | Anterior >> Posterior | Anterior >> Posterior |
| **Bandwidth** | 2894 Hz/Px | 3987 Hz/Px | 2894 Hz/Px | 2298 Hz/Px | 2894 Hz/Px | 2894 Hz/Px | 2604 Hz/Px | 2894 Hz/Px | 2894 Hz/Px |
| **Acquisition order** | Interleaved – Ascending | Ascending | Interleaved - Ascending | Interleaved - Ascending | Interleaved - Ascending | Interleaved - Ascending | Interleaved - Ascending | Interleaved - Ascending | Interleaved - Ascending |
| **Number of slices** | 34 | 34 | 34 | 34 | 34 | 34 | 32 | 34 | 33 |
| **Measurements** | 250 | 250 | 250 | 250 | 250 | 250 | 250 | 250 | 250 |
| **Effective voxel size (mm³)** | 3.3 x 3.3 x 3.0 | 3.3 x 3.3 x 3.0 | 3.3 x 3.3 x 3.0 | 3.3 x 3.3 x 3.0 | 3.3 x 3.3 x 3.0 | 3.3 x 3.3 x 3.0 | 3.3 x 3.3 x 3.0 | 3.3 x 3.3 x 3.0 | 3.3 x 3.3 x 3.0 |
| **Acquisition time (TA)** | 8:24 min | 8:30 min | 8:24 min | 8:37 min | 8:24 min | 8:24 min | 8:24 min | 8:24 min | 8:24 min |

## Functional measurement - DRD Task

| **Site** | **Berlin** | **Bochum** | **Dresden** | **Frankfurt** | **Göttingen** | **Heidelberg** | **Hamburg** | **Marburg** | **Tübingen** |
| --- | --- | --- | --- | --- | --- | --- | --- | --- | --- |
| **Repetition time (TR)** | 1900 ms | 1900 ms | 1900 ms | 1900 ms | 1900 ms | 1900 ms | 1900 ms | 1900 ms | 1900 ms |
| **Echo time (TE)** | 30 ms | 30 ms | 30 ms | 30 ms | 30 ms | 30 ms | 30 ms | 30 ms | 30 ms |
| **Field of View (FoV) mm²** | 192 x 192 | 192 x 192 | 192 x 192 | 192 x 192 | 192 x 192 | 192 x 192 | 192 x 192 | 192 x 192 | 192 x 192 |
| **Matrix size** | 64 x 64 | 64 x 64 | 64 x 64 | 64 x 64 | 64 x 64 | 64 x 64 | 64 x 64 | 64 x 64 | 64 x 64 |
| **Slice thickness** | 3.0 mm | 3.0 mm | 3.0 mm | 3.0 mm | 3.0 mm | 3.0 mm | 3.0 mm | 3.0 mm | 3.0 mm |
| **Distance factor** | 20 % | 20 % | 20 % | 20 % | 20 % | 20 % | 20 % | 20 % | 20 % |
| **Flip angle** | 70° | 70° | 70° | 70° | 70° | 70° | 70° | 70° | 70° |
| **Phase encoding direction** | Anterior >> Posterior | Anterior >> Posterior | Anterior >> Posterior | Anterior >> Posterior | Anterior >> Posterior | Anterior >> Posterior | Anterior >> Posterior | Anterior >> Posterior | Anterior >> Posterior |
| **Bandwidth** | 2894 Hz/Px | 3653 Hz/Px | 2894 Hz/Px | 2298 Hz/Px | 2894 Hz/Px | 2894 Hz/Px | 2232 Hz/Px | 2894 Hz/Px | 2894 Hz/Px |
| **Acquisition order** | Interleaved - Ascending | Interleaved | Interleaved - Ascending | Interleaved - Ascending | Interleaved - Ascending | Interleaved - Ascending | Interleaved - Ascending | Interleaved - Ascending | Interleaved - Ascending |
| **Number of slices** | 31 | 31 | 31 | 31 | 31 | 31 | 30 | 31 | 31 |
| **Measurements** | 185 | 185 | 185 | 185 | 185 | 185 | 185 | 185 | 185 |
| **Effective voxel size (mm³)** | 3.0 x 3.0 x 3.0 | 3.0 x 3.0 x 3.0 | 3.0 x 3.0 x 3.0 | 3.0 x 3.0 x 3.0 | 3.0 x 3.0 x 3.0 | 3.0 x 3.0 x 3.0 | 3.0 x 3.0 x 3.0 | 3.0 x 3.0 x 3.0 | 3.0 x 3.0 x 3.0 |
| **Acquisition time (TA)** | 5:55 min | 6:00 min | 5:55 min | 5:55 min | 5:55 min | 5:55 min | 5:55 min | 5:55 min | 5:55 min |

## Functional measurement - Face Task

| **Site** | **Berlin** | **Bochum** | **Dresden** | **Frankfurt** | **Göttingen** | **Heidelberg** | **Hamburg** | **Marburg** | **Tübingen** |
| --- | --- | --- | --- | --- | --- | --- | --- | --- | --- |
| **Repetition time (TR)** | 2000 ms | 2000 ms | 2000 ms | 2000 ms | 2000 ms | 2000 ms | 2000 ms | 2000 ms | 2000 ms |
| **Echo time (TE)** | 30 ms | 30 ms | 30 ms | 30 ms | 30 ms | 30 ms | 30 ms | 30 ms | 30 ms |
| **Field of View (FoV) mm²** | 210 x 210 | 210 x 210 | 210 x 210 | 210 x 210 | 210 x 210 | 210 x 210 | 210 x 210 | 210 x 210 | 210 x 210 |
| **Matrix size** | 64 x 64 | 64 x 64 | 64 x 64 | 64 x 64 | 64 x 64 | 64 x 64 | 64 x 64 | 64 x 64 | 64 x 64 |
| **Slice thickness** | 3.8 | 3.8 | 3.8 | 3.8 | 3.8 | 3.8 | 3.8 | 3.8 | 3.8 |
| **Distance factor** | 10 % | 10 % | 10 % | 10 % | 10 % | 10 % | 10 % | 10 % | 10 % |
| **Flip angle** | 90° | 90° | 90° | 90° | 90° | 90° | 90° | 90° | 90° |
| **Phase encoding direction** | Anterior >> Posterior | Anterior >> Posterior | Anterior >> Posterior | Anterior >> Posterior | Anterior >> Posterior | Anterior >> Posterior | Anterior >> Posterior | Anterior >> Posterior | Anterior >> Posterior |
| **Bandwidth** | 2232 Hz/Px | 3987 Hz/Px | 2232 Hz/Px | 2298 Hz/Px | 2298 Hz/Px | 2232 Hz/Px | 2232 Hz/Px | 2232 Hz/Px | 2368 Hz/Px |
| **Acquisition order** | Interleaved - Ascending | Interleaved | Interleaved - Ascending | Interleaved - Ascending | Interleaved - Ascending | Interleaved - Ascending | Interleaved - Ascending | Interleaved - Ascending | Interleaved - Ascending |
| **Number of slices** | 33 | 33 | 33 | 33 | 33 | 33 | 32 | 33 | 33 |
| **Measurements** | 187 | 187 | 187 | 187 | 187 | 187 | 187 | 187 | 187 |
| **Effective voxel size (mm³)** | 3.3 x 3.3 x 3.8 | 3.3 x 3.3 x 3.8 | 3.3 x 3.3 x 3.8 | 3.3 x 3.3 x 3.8 | 3.3 x 3.3 x 3.8 | 3.3 x 3.3 x 3.8 | 3.3 x 3.3 x 3.8 | 3.3 x 3.3 x 3.8 | 3.3 x 3.3 x 3.8 |
| **Acquisition time (TA)** | 6:20 min | 6:24 min | 6:20 min | 6:20 min | 6:20 min | 6:20 min | 6:20 min | 6:20 min | 6:18 min |

## Functional measurement - ToM Task

| **Site** | **Berlin** | **Bochum** | **Dresden** | **Frankfurt** | **Göttingen** | **Heidelberg** | **Hamburg** | **Marburg** | **Tübingen** |
| --- | --- | --- | --- | --- | --- | --- | --- | --- | --- |
| **Repetition time (TR)** | 2000 ms | 2000 ms | 2000 ms | 2000 ms | 2000 ms | 2000 ms | 2000 ms | 2000 ms | 2000 ms |
| **Echo time (TE)** | 30 ms | 30 ms | 30 ms | 30 ms | 30 ms | 30 ms | 30 ms | 30 ms | 30 ms |
| **Field of View (FoV) mm²** | 192 x 192 | 192 x 192 | 192 x 192 | 192 x 192 | 192 x 192 | 192 x 192 | 192 x 192 | 192 x 192 | 192 x 192 |
| **Matrix size** | 64 x 64 | 64 x 64 | 64 x 64 | 64 x 64 | 64 x 64 | 64 x 64 | 64 x 64 | 64 x 64 | 64 x 64 |
| **Slice thickness** | 4.0 mm | 4.0 mm | 4.0 mm | 4.0 mm | 4.0 mm | 4.0 mm | 4.0 mm | 4.0 mm | 4.0 mm |
| **Distance factor** | 25 % | 25 % | 25 % | 25 % | 25 % | 25 % | 25 % | 25 % | 25 % |
| **Flip angle** | 80° | 80° | 80° | 80° | 80° | 80° | 80° | 80° | 80° |
| **Phase encoding direction** | Anterior >> Posterior | Anterior >> Posterior | Anterior >> Posterior | Anterior >> Posterior | Anterior >> Posterior | Anterior >> Posterior | Anterior >> Posterior | Anterior >> Posterior | Anterior >> Posterior |
| **Bandwidth** | 2112 Hz/Px | 3653 Hz/Px | 2112 Hz/Px | 2298 Hz/Px | 2112 Hz/Px | 2112 Hz/Px | 2112 Hz/Px | 2112 Hz/Px | 2112 Hz/Px |
| **Acquisition order** | Interleaved - Descending | Interleaved | Interleaved - Descending | Interleaved - Descending | Interleaved - Descending | Interleaved - Descending | Interleaved - Descending | Interleaved - Descending | Interleaved - Descending |
| **Number of slices** | 32 | 32 | 32 | 32 | 32 | 32 | 32 | 32 | 32 |
| **Measurements** | 239 | 239 | 239 | 239 | 239 | 239 | 239 | 239 | 239 |
| **Effective voxel size (mm³)** | 3.0 x 3.0 x 4.0 | 3.0 x 3.0 x 4.0 | 3.0 x 3.0 x 4.0 | 3.0 x 3.0 x 4.0 | 3.0 x 3.0 x 4.0 | 3.0 x 3.0 x 4.0 | 3.0 x 3.0 x 4.0 | 3.0 x 3.0 x 4.0 | 3.0 x 3.0 x 4.0 |
| **Acquisition time (TA)** | 8:02 min | 8:08 min | 8:02 min | 8:02 min | 8:02 min | 8:02 min | 8:04 min | 8:02 min | 8:02 min |

## Fieldmap

| **Site** | **Berlin** | **Bochum** | **Dresden** | **Frankfurt** | **Göttingen** | **Heidelberg** | **Hamburg** | **Marburg** | **Tübingen** |
| --- | --- | --- | --- | --- | --- | --- | --- | --- | --- |
| **Repetition time (TR)** | 531 ms | 531 ms | 531 ms | 540 ms | 531 ms | 531 ms | 531 ms | 531 ms | 531 ms |
| **Echo time (TE) 1** | 4.92 ms | 4.92 ms | 4.92 ms | 4.92 ms | 4.92 ms | 4.92 ms | 4.92 ms | 4.92 ms | 4.92 ms |
| **TE 2** | 7.38ms | 7.38ms | 7.38ms | 7.38ms | 7.38ms | 7.38ms | 7.38ms | 7.38ms | 7.38ms |
| **Field of View (FoV) mm²** | 256 x 256 | 256 x 256 | 256 x 256 | 256 x 256 | 256 x 256 | 256 x 256 | 256 x 256 | 256 x 256 | 256 x 256 |
| **Matrix size** | 78 x 78 | 128 x 128 | 78 x 78 | 64 x 64 | 78 x 78 | 78 x 78 | 78 x 78 | 78 x 78 | 78 x 78 |
| **Slice thickness** | 3.0 mm | 3.0 mm | 3.0 mm | 3.0 mm | 3.0 mm | 3.0 mm | 3.0 mm | 3.0 mm | 3.0 mm |
| **Distance factor** | 10% | 10% | 10% | 10% | 10% | 10% | 10% | 10% | 10% |
| **Flip angle** | 60° | 60° | 60° | 60° | 60° | 60° | 60° | 60° | 60° |
| **Phase encoding direction** | Anterior >> Posterior | Right >> Left | Anterior >> Posterior | Anterior >> Posterior | Anterior >> Posterior | Anterior >> Posterior | Anterior >> Posterior | Anterior >> Posterior | Right >> Left |
| **Bandwidth** | 261 Hz/Px | 290 Hz/Px | 261 Hz/Px | 261 Hz/Px | 261 Hz/Px | 261 Hz/Px | 290 Hz/Px | 261 Hz/Px | 290 Hz/Px |
| **Acquisition order** | Interleaved - Interleaved | default | Interleaved - Interleaved | Interleaved - Interleaved | Interleaved - Interleaved | Interleaved - Interleaved | Interleaved - Interleaved | Interleaved - Interleaved | Interleaved - Interleaved |
| **Number of slices** | 50 | 50 | 50 | 50 | 50 | 50 | 50 | 50 | 50 |
| **Measurements** | 1 | 1 | 1 | 1 | 1 | 1 | 1 | 1 | 1 |
| **Effective voxel size (mm³)** | 3.3 x 3.3 x 3.0 | 3.3 x 3.3 x 3.0 | 3.3 x 3.3 x 3.0 | 4.0 x 4.0 x 3.0 | 3.3 x 3.3 x 3.0 | 3.3 x 3.3 x 3.0 | 3.3 x 3.3 x 3.0 | 3.3 x 3.3 x 3.0 | 3.3 x 3.3 x 3.0 |
| **Acquisition time (TA)** | 1:25 | 1:25 | 1:25 | 1:12 | 1:25 | 1:25 | 1:25 | 1:25 | 1:25 |
